# Diversidad genética y resistencia antirretroviral del virus de la inmunodeficiencia humana de tipo 1 en personal militar peruano a los 12 y a los 18 meses de seguimiento

**DOI:** 10.7705/biomedica.7970

**Published:** 2026-06-01

**Authors:** Jonathan Guerrero, Dilan Suárez, Carlos Augusto Yabar

**Affiliations:** 1 Laboratorio de Biomedicina y Microbiología, Instituto Nacional de Salud, Lima, Perú Laboratorio de Biomedicina y Microbiología Instituto Nacional de Salud Lima Perú; 2 Laboratorio de Virus de Transmisión Sexual, Unidad de Virología, Instituto Nacional de Salud, Lima, Perú Laboratorio de Virus de Transmisión Sexual Instituto Nacional de Salud Lima Perú; 3 Facultad de Medicina Humana, Universidad de San Martín de Porres, Lima, Perú Facultad de Medicina Humana Universidad de San Martín de Porres Lima Perú

**Keywords:** VIH, conducta sexual, antirretrovirales, evolución molecular, variación genética, Perú, HIV, sexual behaviour, anti-retroviral agents, evolution, molecular, genetic variation, Perú

## Abstract

**Introducción.:**

Se desconocen los factores que afectan la diversidad genética y las mutaciones de resistencia al tratamiento antirretroviral del virus de la inmunodeficiencia humana en personal militar.

**Objetivo.:**

Describir los factores que afectan la variabilidad genética y la resistencia a los antirretrovirales del virus de la inmunodeficiencia humana en población militar durante un año de seguimiento.

**Materiales y métodos.:**

Se incluyeron 52 militares peruanos infectados con el virus de la inmunodeficiencia humana y con terapia antirretroviral. Luego, se comparó la diversidad genética, los subtipos genéticos, los eventos de recombinación y el perfil de resistencia que ocurrieron en los genes de la proteasa y la transcriptasa inversa durante 12 y 18 meses de seguimiento, considerando las características socio-epidemiológicas de los participantes.

**Resultados.:**

Se observó uso intermitente del preservativo (37 %), contacto con trabajadoras sexuales (17 %) y antecedentes de infecciones de transmisión sexual (56 %). La tasa de diversidad genética (n) se elevó de 0,30 a 0,45, mientras que los subtipos genéticos y formas recombinantes no cambiaron significativamente. De 11 muestras que evidenciaron cambios significativos de nucleótidos, 5 presentaron mutaciones de resistencia. Finalmente, el análisis bivariado demostró que a mayor edad de los sujetos, mayor asociación significativa con una diferencia del perfil de resistencia después de un intervalo de 12 meses o más.

**Conclusión.:**

Se demuestra que después de 12 y 18 meses de seguimiento, el virus de la inmunodeficiencia humana que infecta un grupo de personal militar peruano sufrió cambios de su diversidad genética y de perfil de resistencia, los cuales se asociaron con la edad de los participantes.

## Abstract (en)

**Introduction.:**

The factors affecting genetic diversity and antiretroviral therapy resistance mutations in human immunodeficiency virus (HIV) type 1 in military personnel are unknown.

**Objective.:**

To describe the factors affecting genetic variability and resistance to HIV antiretrovirals in the military population during one year of follow-up.

**Materials and methods.:**

Fifty-two Peruvian military personnel living with HIV and receiving antiretroviral therapy were recruited. Genetic diversity, genetic subtypes, recombination events, and resistance profile occurring in protease and reverse transcriptase genes were compared at 12 and 18 month follow-up, taking into account the socio-epidemiological characteristics of the participants.

**Results.:**

Intermittent condom use (37%), contact with sex workers (17%) and a history of sexually transmitted infections (56%) were observed. The genetic diversity index (n) increased from 0.30 to 0.45, while genetic subtypes and recombinant forms did not change significantly. Of eleven samples with significant nucleotide changes, five showed resistance mutations. Finally, bivariate analysis showed that increasing subject age was significantly associated with a difference in resistance profile after an interval of 12 months or more.

**Conclusions.:**

We show that, after at 12 and 18 month of follow-up, HIV-infecting a population of Peruvian military subjects underwent changes in its genetic diversity and resistance profile, which were associated with the chronological age of the participants.

HIV/sida es un problema de salud pública que afecta a más de 23 millones de personas en todo el mundo, cobrando la vida de cerca de 40,4 millones de personas ^1^. El surgimiento y desarrollo de la terapia antirretroviral ha permitido mejorar la calidad de vida de estos pacientes ^2^, reduciendo significativamente la tasa de mortalidad de la enfermedad ^3^, principalmente en los países con mayor prevalencia. Sin embargo, el incremento de la resistencia pretratamiento y la resistencia adquirida ha afectado significativamente la efectividad de la terapia antirretroviral, provocando el surgimiento de cepas resistentes de HIV ^4^.

En Perú, la infección por HIV/sida continúa siendo un importante problema de salud pública, con una prevalencia del 0,23 % en la población general, concentrada principalmente en la población de hombres que tienen sexo con otros hombres y de mujeres trabajadoras sexuales ^5^.

En el 2008, el Ministerio de Salud de Perú estableció la prueba de genotipificación de HIV para la vigilancia de la resistencia a nivel nacional ^6^. Gracias a esta prueba, no solo se ha logrado caracterizar la resistencia adquirida y transmitida del HIV en este país ^7^^,^^8^, sino también identificar los nuevos subtipos y formas recombinantes que circulan en los sujetos con comportamiento sexual de riesgo ^9^^,^^10^.

Con relación a este último punto, diferentes estudios han comparado los genotipos de HIV que circulan entre los grupos con conducta sexual de riesgo, demostrando que esta característica no solo afecta la diversidad genética del HIV ^11^^-^^13^ sino también el perfil de resistencia a la terapia antirretroviral ^9^^,^^14^^-^^17^. Esto, probablemente, se debe a que la conducta sexual de riesgo promueve la transmisión de variantes genéticas de manera diferencial, trayendo como consecuencia la generación de formas recombinantes circulantes ^18^. Estas formas tienen un gran impacto epidemiológico, porque son capaces de combinar las características de resistencia a la terapia antirretroviral proveniente de las especies de HIV-1 que le dieron origen, lo que genera la aparición de cepas resistentes ^19^^,^^20^.

Si bien algunos estudios sobre análisis filogenético y epidemiología molecular han revelado que existen grupos humanos con conducta de alto riesgo, tales como hombres homosexuales, mujeres transgénero, trabajadoras sexuales femeninas e incluso, usuarios de drogas intravenosas ^16^^,^^17^^,^^21^^,^^22^ que cumplen un rol importante en la transmisión del virus, existen muy pocos estudios realizados a partir de personal militar.

Esta población, en particular, se caracteriza por su asociación con factores psicosociales tales como trauma, abuso sexual y preponderancia por demostrar su masculinidad, lo que, en su conjunto, conduce también a prácticas sexuales de alto riesgo de infección por HIV ^23^^-^^25^. Estas características convierten a la población militar en reservorios epidemiológicos críticos capaces de facilitar la introducción y circulación de nuevas variantes genéticas y formas recombinantes del HIV, así como a contribuir a la diseminación de genotipos resistentes a la terapia antirretroviral.

En concordancia con los estudios socio-epidemiológicos, diferentes investigaciones a nivel molecular revelaron una alta frecuencia de contagios con HIV del subtipo B y diferentes formas recombinantes de HIV en sujetos militares destacados a diferentes países o que tuvieron contacto sexual con trabajadoras sexuales femeninas ^26^^-^^30^. Sin embargo, estos estudios corresponden a países cuyas características culturales y socio-epidemiológicas difieren de la realidad peruana.

Asimismo, nuestro equipo de investigación realizó un estudio piloto a partir de una población de 11 militares peruanos, con el fin de conocer su perfil de resistencia molecular del HIV y los subtipos circulantes, demostrando que los 11 pacientes estaban infectados con HIV del subtipo B, con la presencia de dos casos de resistencia a los inhibidores de proteasa y los inhibidores nucleósidos de la transcriptasa inversa ^31^. Si bien este estudio fue el primero en corroborar la variante genética y el patrón de resistencia que afectó a un grupo de militares peruanos, es probable que, después de casi 20 años, nuevas variantes genéticas y genotipos resistentes podrían estar siendo introducidas en el país.

Además, la evidencia molecular disponible en sujetos militares es escasa, ya que los estudios publicados sobre militares peruanos son limitados en tamaño y diseño (transversal) que, si bien brinda una mirada amplia de las características genéticas del virus, no nos permite hacer un seguimiento a lo largo del tiempo que ayude a determinar las nuevas variantes, las formas recombinantes o los genotipos resistentes que van apareciendo, en concordancia con la descripción de la actual conducta sexual de riesgo.

En ese sentido, el presente estudio plantea un análisis longitudinal a partir de un grupo de militares peruanos para determinar si, en un intervalo de tiempo de aproximadamente 1 año, el HIV sufre cambios genéticos significativos, y si estos cambios se ven afectados por la presencia de nuevos subtipos, formas recombinantes, eventos de cuellos de botella, perfil de resistencia o por las características socio-epidemiológicas de los sujetos de investigación.

## Materiales y métodos

El estudio contempló la inclusión de 52 militares de sexo masculino, mayores de 18 años, provenientes del Hospital Militar Central (Lima, Perú), quienes firmaron un consentimiento informado para participar en el estudio.

El estudio y el documento de consentimiento informado fueron aprobados por el Comité Institucional de Ética en Investigación del Instituto Nacional de Salud del Perú, bajo el código OT-033-18. La primera toma de muestras de los participantes se hizo entre marzo del 2015 y noviembre del 2017, y la segunda, entre junio del 2016 y marzo del 2018.

El estudio siguió un diseño longitudinal con tomas de muestras a los 12 y a los 18 meses, considerando lo siguiente:

*Criterios de inclusión*

- Personal militar en actividad o en retiro, perteneciente a cualquiera de los tres institutos armados del Perú (Ejército, Marina o Fuerza Aérea),
- diagnóstico confirmado de infección por HIV-1,
- mayores de 18 años,
- participación voluntaria y firma del consentimiento informado,
- suficiente cantidad y calidad de plasma o sangre total y
- disponibilidad, al menos, de dos muestras seriadas con un intervalo aproximado de un año para permitir el análisis longitudinal.

*Criterios de exclusión*

- Sujetos con tratamientos antirretrovirales interrumpidos en los últimos seis meses antes de la segunda toma de muestra,
- participantes que no completen el seguimiento longitudinal o que no cuenten con una segunda muestra,
- sujetos con datos socio-epidemiológicos incompletos, esenciales para correlacionar las conductas de riesgo con los análisis moleculares y
- participantes que rechacen continuar en el estudio en cualquier momento.

### Recolección de la muestra

Se tomó por triplicado un volumen de 6 ml de sangre venosa obtenido del antebrazo derecho o izquierdo mediante punción, utilizando una aguja hipodérmica insertada a un tubo de extracción de sangre al vacío con anticoagulante (EDTA). La sangre fue almacenada a temperatura ambiente en cajas de tecnopor que luego se trasladaron al Instituto Nacional de Salud de Perú para su registro y codificación en el área de recepción y obtención de la muestra.

Los tres tubos de sangre con 6 ml de cada paciente fueron empleados para diferentes procedimientos. El primer tubo se utilizó para determinar la carga viral; el segundo se procesó para el recuento de linfocitos CD4.

El tercer tubo de sangre se utilizó para los procedimientos moleculares, para lo cual la muestra fue separada en dos partes: la primera, de 1 ml, para el análisis de HIV proviral de sangre total, el cual fue colocado en un criovial debidamente rotulado y almacenado a -80 °C. La segunda se utilizó para la separación del plasma y subsecuente análisis de HIV circulante en sangre. Para tal efecto, la sangre se centrifugó a 4000 RPM por 10 minutos a temperatura ambiente. El sobrenadante fue dividido en alícuotas de 1,5 ml en dos crioviales de 2 ml y se almacenó a -80 °C hasta su posterior uso.

### Extracción del material genético

Para la extracción del ARN o ADN proviral, se tomaron 200 μl de sangre periférica o plasma siguiendo las instrucciones recomendadas por el fabricante del kit comercial Thermo Scientific™ GeneJET. La muestra fue sometida a una solución de lisis para la liberación del material genético, seguida de lavados con etanol al 70 % y soluciones tampón de lavado.

El ácido nucleico fue removido de las columnas de purificación provistas por el kit mediante centrifugación utilizando un tampón con inhibidor de ARNasas como diluyente. Los crioviales con el material genético se almacenaron a -80 °C para los análisis siguientes. Además, se incluyó como ADN de control de la PCR a la cepa de referencia HXB2, subtipo B.

### Reacción en cadena de la polimerasa

Para la amplificación del gen *gag* se siguieron las especificaciones descritas anteriormente 30, que constan de dos rondas de amplificación. En la primera ronda, se preparó la mezcla de reacción que contenía concentraciones finales de los siguientes componentes: solución tampón de reacción de PCR II (10 mM Tris-HCl, 50 mM KCl, pH 8,3); 2,5 mM de MgCl_2_; 0,8 mM de mezcla de trifosfatos de deoxinucleótidos (200 μM de dATP, dGTP, dCTP y dTTP); 0,2 pmoles de los cebadores H1G777 y H1P202; 0,25 U de enzima Taq ADN polimerasa (Applied Biosystem™) y 100 ng de ADN molde. Las condiciones de los ciclos fueron: 35 ciclos de 94 °C por 30 s, 50 °C por 30 s y 72 °C por 2 minutos y 1 ciclo final de 72 °C por 7 minutos y, por último, una temperatura de almacenaje de 4 °C.

En la segunda ronda de PCR anidada *(nested* PCR), se mezclaron concentraciones finales de los siguientes reactivos: solución tampón de reacción de PCR (10 mM Tris-HCl, 50 mM KCl, pH 8,3); 2,5 mM de MgCl_2_; 0,8 mM de mezcla de trifosfatos de deoxinucleótidos (200 μM de dATP, dGTP, dCTP y dTTP); 0,4 pmoles de los cebadores H1Gag1584 y g17; 0,25U de enzima Taq ADN polimerasa (Applied Biosystemji) y 2 μl de reacción de la primera ronda.

Las condiciones de los ciclos fueron: tres ciclos iniciales de 94 °C por 1 minuto, 50 °C por 1 minuto y 72 °C por 1 minuto, seguido de 32 ciclos de 94 °C por 30 s, 45 °C por 30 s y 72 °C por 2 minutos. Se realizó una extensión final de 72 °C por 7 minutos y, finalmente, se programó una temperatura de almacenaje a 4 °C.

Para la amplificación del gen *pol (prt-tr)* se siguieron los procedimientos que reportamos anteriormente ^10^, los cuales consistieron en un ensayo de PCR y PCR anidada para la amplificación de un fragmento de 1700 pb (primera ronda) y de 1500 pb (segunda ronda).

En la primera ronda, se preparó una mezcla con concentraciones finales de los siguientes componentes: solución tampón de reacción 1X (10 mM Tris-HCl, 50 mM KCl, pH 8,3); 1,5 mM de MgCl_2_; 0,8 mM de mezcla de dNTP (200 μM de dATP, dGTP, dCTP y dTTP); 0,5 pmol del cebador RT-21; 0,5 pmol del cebador 1243; 1 pmol del cebador MAW-26 y 1,25 U de enzima Taq polimerasa. Las condiciones de amplificación fueron las siguientes: 1 ciclo inicial de desnaturalización de 94 °C por 2 minutos, 40 ciclos de 94 °C por 15 s, 55 °C por 20 s, 72 °C por 2 minutos y un ciclo de extensión final de 72 °C por 10 minutos.

En la segunda ronda de PCR, se utilizaron concentraciones finales de los siguientes reactivos: solución tampón de reacción 1X (10 mM Tris-HCl, 50 mM KCl, pH 8,3); 1,5 mM de MgCl_2_; 0,8 mM de mezcla de dNTPs (200 μM de dATP, dGTP, dCTP y dTTP); 0,2 pmol del cebador PRO-1, 0,1 pmoles de RT-20 y 0,1 pmoles de 1205 y 1,25 U de enzima Taq polimerasa.

Las condiciones de amplificación fueron las siguientes: desnaturalización inicial de 94 °C por 2 minutos, 35 ciclos de 94 °C por 15 s, 63 °C por 20 s y 72 °C por 2 minutos, una extensión final de 72 °C por 10 minutos y, finalmente, una temperatura de almacenaje de 4 °C. Todos los productos de PCR fueron visualizados por electroforesis, siendo de 460 pb para el gen *gag* y de 1200 pb para *pol.*

### Secuenciación de ADN

Se secuenciaron los genes *gag* y *pol* utilizando el analizador genético ABI 3500 XL y el kit BigDye Terminator™, versión 3.1, Cycle Sequencing. Para tal propósito, se añadieron 10 ng de ADN en una mezcla de 20 μl de reacción que contenía 4 μl de BigDye, 1X de la solución tampón de secuenciación BigDye y 0,25 pmol de cebadores.

Para la secuenciación de la región *gag,* se utilizaron los cebadores. H1Gag1584 y g17, y para *pol (prt-tr),* los cebadores. MAW-46, DSPR, PSR2, RT-a, RT-y, RT-b y HXB2-89.

La reacción de secuenciación se sometió a 96 °C por 1 minuto, 25 ciclos de 96 °C por 10 s, 50 °C por 5 s y 60 °C por 4 minutos. Luego, los productos se mezclaron con una solución de precipitación fría que contenía 3M de acetato de sodio de pH 5,4 y 78 % de etanol. La mezcla se incubó entre 20 °C y 25 °C por 20 minutos y luego se centrifugó a 2000g por 30 minutos. Después de un proceso de lavado con 70 % de alcohol, la mezcla se centrifugó a 2000g por 15 minutos y los productos se secaron entre 20 °C y 25 °C por 20 minutos. A continuación, los productos de secuenciación se desnaturalizaron con 15 μl de formamida pura y 95 °C por 2 minutos. Finalizada la desnaturalización, se colocó la placa en el analizador genético ABI 3500 XL.

### Determinación de la carga viral y recuento de CD4-CD8

Para la determinación de la carga viral, se realizó una prueba de PCR en tiempo real usando el equipo COBAS Ampliprep™ y COBAS Taqman HIV-1™ (Roche) mientras que para el recuento de CD4-CD8-CD3 se llevó a cabo por citometría de flujo usando un equipo FACSCoun™ y FACSCanto II™ (Beckton Dickinson).

### Análisis bioinformático

Se utilizó el programa SeqScape, versión 2.7, para el análisis y ensamblaje de las secuencias. Los consensos se exportaron en formato FASTA para su posterior alineamiento y construcción de los árboles filogenéticos en el programa MEGA, versión 10.1.

Para la selección del modelo de sustitución nucleotídica, se recurrió al *software* JmodelTest, versión 2.1.10, el cual mostró al modelo GTR+G como el más apropiado para el análisis de ambos genes.

Para la concatenación de los genes *gag-pol,* se recurrió al programa BioEdit, versión 7.2.5 (https://bioedit.software.informer.com/7.2/), el cual permitió determinar la diversidad genética entre el primer (T_1_) y el segundo tiempo (T_2_) (T_1_ frente a T_2_).

Para la determinación de los eventos de recombinación, se utilizaron las plataformas *website* COMET HIV-1, versión 2.2 (https://comet.lih.lu/index.php?cat=hiv1), REGA HIV-1, versión 3.0 (http://dbpartners.stanford.edu:8080/RegaSubtyping/stanford-hiv/typingtool/), jpHMM HIV-1 (http://jphmm.gobics.de/submission_hiv) y RIP, versión 3.0 (https://www.hiv.lanl.gov/content/sequence/RIP/RIP.html).

Para la determinación del perfil de resistencia, se recurrió al programa *HIV Drug Resistance* de Stanford University (https://hivdb.stanford.edu/). Las mutaciones asociadas a la resistencia fueron identificadas utilizando el algoritmo de interpretación del mencionado programa, con base en los listados de resistencia de la *International Antiviral Society* de Estados Unidos y de la Organización Mundial de la Salud (WHO *HIV Drug Resistance List),* los cuales constituyen los estándares internacionales para diferenciar las mutaciones de resistencia relevantes de los polimorfismos naturales.

### Análisis estadístico

Se recurrió a la prueba de la tasa relativa de Tajima ^32^^,^^33^ para el análisis de la diversidad genética a nivel de nucleótido (n), el número de cambios no sinónimos y sinónimos (dN-dS), la tasa de transversiones y transiciones y el cálculo de la D de Tajima para la determinación del equilibrio neutral de evolución.

Se realizó la prueba exacta de Fisher mediante análisis bivariado para el cálculo de X^2^ y de p. Asimismo, se hizo el análisis de varianza (ANOVA) para determinar la frecuencia de la resistencia a los antirretrovirales en ambos intervalos de tiempo.

## Resultados

Se incluyeron 52 participantes, de los cuales, el 80,8 % (42/52) tenía más de 40 años; 45 (86,5 %) declararon ser heterosexuales, 3 (5,8 %), bisexuales y 1 (1,9 %), homosexual. Asimismo, el 82,7 % (43/52) negó haber tenido contacto sexual con trabajadoras sexuales, el 67,3 % (35/52) afirmó usar siempre el preservativo y el 19,2 % (10/52) manifestó haber contraído alguna infección de transmisión sexual (cuadro 1).

**Cuadro 1:** Características socio-epidemiológicas, inmunológicas y virológicas de los militares peruanos de Lima y Callao durante el período 2015-2018

Característica		n	%
Edad (años)			
	< 30	3	5,8
	30-39	7	13,5
	40-50	16	30,8
	>50	26	50,0
Tiempo desde el diagnóstico de HIV (años)			
	0 a 10	23	44,2
	11 a 20	18	34,6
	> 20	3	5,8
	Sin dato	8	15,4
Sexo			
	Masculino	52	100,0
	Femenino	0	0,0
Comportamiento sexual			
	Heterosexual	45	86,5
	Homosexual	1	1,9
	Bisexual	3	5,8
	Sin datos	3	5,8
Contacto sexual con trabajadoras sexuales			
	Sí	8	15,4
	No	43	82,7
	Sin datos	1	1,9
Consumo de drogas recreativas			
	Sí	1	1,9
	No	50	96,2
	Sin datos	1	1,9
Uso del preservativo			
	Siempre	35	67,3
	Algunas veces	7	13,5
	Nunca	0	0,0
	Sin datos	10	19,2
Presencia de otras infecciones de transmisión sexual			
	Sí	10	19,2
	No	29	55,8
	Sin datos	13	25,0
Cambio de tratamiento antirretrovirales			
	Sí	13	25,0
	No	39	75,0
Recuento de CD4 (células/ul)			
	T_1_	532,29	NA
	T_2_	580,3	NA
Carga viral (copias/ml)			
	T_1_	3905,32	NA
	T_2_	5262,29	NA

NA: no aplica

Durante T_1_, el 19,2 % (10/52) de los participantes tuvo viremia cuantificable, de los cuales 8 superaron el umbral de más de 200 copias/ ml (criterio clínico de falla virológica); en contraste, en T_2_ se evidenció una mejoría sustancial, ya que solo el 11,5 % (6/52) de los militares tenía carga viral detectable, de los cuales 3 tenían valores compatibles con falla virológica.

### Diversidad genética

De las muestras recolectadas (N = 52), se lograron amplificar y secuenciar 46 (88 %) muestras de HIV correspondiente a la región *gag-pol.* A partir de este grupo, se analizó la diversidad genética de la población de HIV entre T_1_ y T_2_ de la recolección de muestras, de la región concatenada *gag-pol,* utilizando la prueba D de Tajima (cuadro 2).

**Cuadro 2:** Parámetros de diversidad genética de los marcadores *gag* y *pol* del HIV en ambos tiempos

Tiempo	Fragmento analizado	m	S n	dN/dS	Transc/ Transv	D
Primer tiempo (T_1_)	*gag-pol*	46	226 0,030833	0,252232	0,057391	-1,690728
Segundo tiempo (T_2_)	*gag-pol*	46	201 0,045609	0,36612	0,083305	-1,650451

m: número de secuencias; S: número de sitios segregantes; dN/dS: tasa entre cambios no sinónimos (dN) y sinónimos (dS); Transc/Transv: tasa entre transiciones y transversiones, n: diversidad a nivel de nucleótidos; D: prueba de Tajima

De acuerdo con los resultados, se identificó un incremento de la tasa de diversidad genética (n) de 0,30 a 0,45, en los índices de cambios no sinónimos y sinónimos (dN-dS) de 0,25 a 0,36 y en la tasa de transversiones y transiciones de 0,057 a 0,083. El valor de la D de Tajima para ambos tiempos fue negativo, y también se observó un incremento de -1,69 a -1,65 entre T_1_ y T_2_.

**Figura 1:**
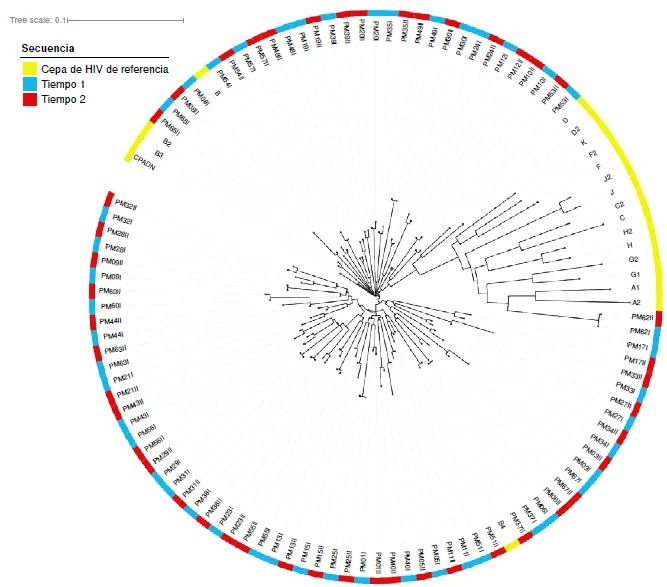
Árbol filogenético de los genes concatenados (Gag-Pol) del HIV-1

Por otro lado, se hizo el análisis filogenético para comparar los subtipos genéticos de HIV entre los dos tiempos establecidos. Según la distribución de los clados filogenéticos, las muestras de HIV entre T_1_ y T_2_ formaron conglomerados *(clusters)* monofiléticos o con estrecha relación filogenética, por lo que no se observó ningún evento de infección mixta de HIV en la población estudiada. Asimismo, el 99 % (51/52) de las muestras de HIV se identificaron como de subtipo B; sin embargo, se detectó que la muestra con el código PM62 mostraba relación filogenética con una cepa de referencia del subtipo A (figura 1).

Un siguiente análisis mediante el aplicativo REGA HIV *Subtyping tool* de Stanford University permitió la identificación de eventos de recombinación intergénico *(gag* y *pol)* e intragénico *(gag* o *pol),* y se hallaron formas recombinantes de tipo BD, BF y AG (cuadro 3). En general, se identificaron seis formas recombinantes en T_1_ y cinco en T_2_.

**Cuadro 3:** Casos de HIV que presentaron recombinación en el intervalo de tiempo inicial (T1) y final (T2)

Código	Subtipo según análisis filogenético	Evento de recombinación		Subtotal recombinaciones n (%)	
		T_1_	T_2_	T_1_	T_2_
PM008		B/F	B/F	1	1
PM010	B	-	B/D	0	1
PM013	B	-	B/F	0	1
PM019	B	B/F	-	1	0
PM023	B	B/F	-	1	0
PM024	B	-	B/D	0	1
PM043	B	-	B/D	0	1
PM049	B	B/F	-	1	0
PM062	A	Formas recombinantes circulantes 02_AG	Formas recombinantes circulantes 02_AG	1	1
Total, recombinaciones T_1_: n (%)				5 (10,9)	-
Total, recombinaciones T_2_: n (%)				-	6 (13,0)

En T_2_ se observó que tres formas recombinantes revirtieron al subtipo B puro, mientras que cuatro aparecieron *de novo* y dos cepas de HIV se mantuvieron en su forma recombinante original.

### Mutaciones de resistencia

En T_1_, se observó que L90M era la mutación más frecuente (4,3 %), asociada con la resistencia a múltiples inhibidores de proteasas, mientras que en T_2_, las mutaciones principales fueron D30N (6,5 %) y, nuevamente, L90M (4,3 %).

Por otro lado, para los inhibidores no nucleósidos de la transcriptasa inversa se destaca la mutación K103K/N (6,5 %) en T_1_, que confiere resistencia de alto nivel a efavirenz y nevirapina; para T_2_ se detectaron K103N y P225P/H (4,3 % cada una).

Finalmente, para los inhibidores nucleósidos de la transcriptasa inversa, en T, se identificaron las mutaciones L210L/W y M184V (8,7 % cada una), mientras que en T_2_ se volvió a detectar M184V (8,7 %) y D67N (6,5 %).

Todas las mutaciones mencionadas como resistentes corresponden a variantes establecidas en las listas oficiales (IAS-USA, WHO, Stanford HIVdb) y no se consideran polimorfismos naturales del subtipo B, predominante en la población militar.

### Análisis de divergencia evolutiva

**Figura 2:**
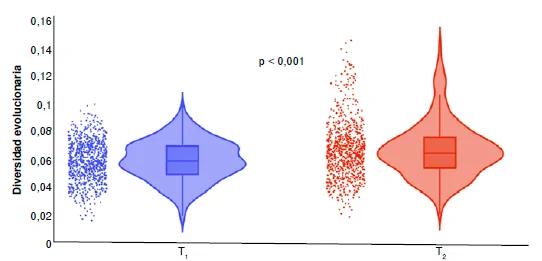
Gráfico en violin que muestra las estimaciones de la divergencia evolutiva sobre pares de secuencias dentro de grupos entre el tiempo 1 (T_1_) y tiempo 2 (T_2_). Los análisis evolutivos se realizaron utilizando MEGA (versión 6) y visualización mediante la herramienta de gráficos en linea de Plotly (https://chart-studio.plot.ly). Las estadísticas de comparación del grupo se calcularon mediante la prueba U de Mann-Whitney.

Se determinó la divergencia evolutiva de las muestras entre los pares de secuencias de los grupos analizados entre T_1_ y T_2_. De acuerdo con los datos obtenidos de la prueba U de Mann-Whitney, la divergencia evolutiva de la población analizada entre ambos tiempos fue significativa (p < 0,01) (figura 2). Asimismo, se hizo la comparación de los cambios de nucleótidos de cada uno de los casos entre T_1_ y T_2_. De acuerdo con los resultados, 8 de 46 (17,4 %) muestras presentaron divergencias significativas (cuadro 4).

**Cuadro 4:** Análisis de cambios de nucleótidos de las muestras de HIV de militares peruanos entre T_1_ y T_2_

		**Genes concatenados *gag-pol***						
N°	Código	Tiempo 1	Tiempo 2	Cepa HXB289	Divergencia	Sitios idénticos	X^2^	p
1	PM001	6	5	37	0	1429	0,09	0,76302
2	PM003	6	10	59	1	1299	1,00	0,31731
3	PM005	0	34	66	0	1498	34,00	**0,00000**
4	PM006	0	0	98	0	1522	0,00	1,00000
5	PM008	5	21	45	0	1374	9,85	**0,00170**
6	PM009	0	1	77	0	1395	1,00	0,31731
7	PM010	1	2	44	0	1266	0,33	0,5637
8	PM011	1	7	46	0	1470	4,50	**0,03389**
9	PM012	2	2	39	0	1556	0,00	1,00000
10	PM013	1	5	35	0	1426	2,67	0,10247
11	PM015	2	8	48	0	1416	3,60	0,05778
12	PM017	13	12	64	1	1415	0,04	0,84148
13	PM019	7	10	34	0	1323	0,53	0,46685
14	PM020	5	1	62	1	1373	2,67	0.10247
15	PM021	7	16	36	0	970	3,52	0,06057
16	PM023	1	4	57	1	1534	1,80	0,17971
17	PM024	7	4	64	1	1418	0,82	0,36571
18	PM025	6	2	93	0	1509	2,00	0,1573
19	PM027	7	5	51	0	1375	0,33	0,5637
20	PM028	15	19	52	0	1444	0,47	0,49272
21	PM029	0	0	88	0	1416	0,00	1,00000
22	PM030	0	4	47	0	1439	4,00	**0,0455**
23	PM031	0	1	63	0	1368	1,00	0,31731
24	PM032	9	4	77	0	1362	1,92	0,16552
25	PM033	2	45	83	0	1488	39,34	**0.00000**
26	PM034	10	5	43	1	1199	1,67	0,19671
27	PM035	3	6	66	0	1514	1,00	0,31731
28	PM037	0	3	82	1	1497	3,00	0,08326
29	PM038	5	6	55	1	1366	0,09	0,76302
30	PM039	7	6	61	0	1427	0,08	0,78151
31	PM040	4	17	57	1	1517	8,05	**0,00456**
32	PM043	9	4	72	0	1484	1,92	0,16552
33	PM044	3	24	58	1	1319	16,33	**0,00005**
34	PM048	5	3	36	0	1483	0,50	0,4795
35	PM049	0	1	85	0	1463	1,00	0,31731
36	PM051	7	2	43	0	1532	2,78	0,09558
37	PM053	1	2	64	0	1510	0,33	0,5637
38	PM054	5	2	42	1	1052	1,29	0,25684
39	PM055	3	1	71	0	1498	1,00	0,31731
40	PM056	7	16	73	0	1467	3,52	0,06057
41	PM057	7	2	57	0	1406	2,78	0,09558
42	PM060	0	5	91	0	1496	5,00	0,**02535**
43	PM062	3	4	146	1	1317	0,14	0,70546
44	PM063	2	8	50	1	1521	3,60	0,05778
45	PM065	6	3	53	0	1507	1,00	0,31731
46	PM067	8	4	56	2	1472	1,33	0,24821

Nota: los códigos y valores de p de las muestras con diferencias significativas de diversidad genética a nivel de nucleótidos entre T_1_ y T_2_ se resaltan en negrita.

### Análisis de frecuencia de mutaciones y perfil de resistencia del VIH

Se hizo un análisis de frecuencias respecto a los cambios de mutaciones de resistencia, recombinación, cambios en el perfil de resistencia y casos de disminución de linfocitos CD4 entre T_1_ y T_2_ (cuadro 5). Para ello, se calculó la frecuencia de estas variables entre el grupo de muestras que presentaron cambios significativos de nucleótidos en comparación con las demás. De acuerdo con los resultados obtenidos, se observó que el grupo de muestras que presentaron cambios significativos de nucleótidos tenian una mayor frecuencia de cambios de mutaciones de resistencia y del perfil de resistencia (62,5 %) (5/8) que las demás muestras de HIV, mientras que el 28,1 % (9/32; n = 9) de las muestras que no presentaron cambios significativos de nucleótidos experimentaron una disminución de los valores de linfocitos CD4.

**Cuadro 5:** Comparación de los casos con cambios significativos y no significativos de nucleótidos entre T_1_ y T_2_ en función de los eventos de recombinación, mutaciones de resistencia y disminución del recuento de CD4

Tipo de caso	Total de mutaciones de resistencia a terapia antirretroviral:	Eventos de recombinación n (%)	Cambios en el perfil de resistencia	Casos con disminución de linfocitos CD4
	n (%)		n (%)	n (%)
Significativo (p < 0,05) (n = 8)	5 (62,50)	0	5 (62,50)	0
No significativo (p > 0,05) (n = 32)	10 (31,25)*	6 (18,75)*	9 (28,13)*	9 (28,13)**

* Se excluyeron del análisis 5 muestras sin resultado de secuenciación del gen la transcriptasa inversa.

** Se excluyeron del análisis 2 muestras sin resultados de recuento de linfocitos CD4.

Para el análisis del perfil de resistencia por familias de inhibidores del HIV-1, se demostró que los inhibidores nucleósidos de la transcriptasa inversa presentaron las frecuencias más elevadas de resistencia en los dos tiempos evaluados. Dentro de este grupo, el abacavir fue el antirretroviral con mayor proporción de resistencia, y se incrementó del 21,7 % en T_1_ al 23,9 % en T_2_.

Le siguieron la lamivudina y la emtricitabina, ambas con una frecuencia del 21,7 % en los dos tiempos.

Para el caso de los inhibidores no nucleósidos de la transcriptasa inversa, la nevirapina registró el mayor nivel de resistencia con el 17,4 % en T_1_, y disminuyó al 15,2 % en T_2_; el efavirenz presentó una resistencia del 15,2 % en los dos momentos del estudio.

Finalmente, entre los inhibidores de proteasas se observó un incremento general de resistencia en T_2_, con excepción de dos fármacos: el nelfinavir, cuya resistencia descendió del 13 al 8,7 % y el tipranavirritonavir, que permaneció estable en el 4,3 % para los dos tiempos.

### *Asociación de factores socio-epidemiológicos y virales en T*_*1*_ *y T*_*2*_

Se hizo un análisis bivariado con el fin de conocer si algún factor socio-epidemiológico se asociaba con el polimorfismo genético de nucleotidicos o con los cambios en el nivel de resistencia entre los dos tiempos analizados. De acuerdo con los datos del cuadro 6, hubo cambios significativos del perfil de resistencia del HIV a los 12 y a los 18 meses de seguimiento, según la edad de los participantes, principalmente en aquellos sujetos con más de 50 años.

**Cuadro 6:** Análisis de asociación de las variables socio-epidemiológicas con la diversidad nucleotidica y el perfil de resistencia de HIV entre el tiempo 1 y el tiempo 2

		Total	Diferencia de nucleótidos en dos tiempos		p*	Diferencia del perfil de resistencia en dos tiempos		p*
			(n)			(n)		
			No	Sí		No	Sí	
Edad (años)					No significativo			< 0,01
	< 30	3	2	1		3	0	
	30 - 39	6	6	0		5	1	
	40-50	18	13	5		15	3	
	> 50	18	13	5		6	12	
Comportamiento sexual		43	33	10	No significativo	29	14	No significativo
	Bisexual	2	2	0		2	0	
	Heterosexual	40	30	10		27	13	
	Homosexual	1	1	0		0	1	
Contacto sexual con trabajadoras sexuales		46	35	11	No significativo	30	16	No significativo
	Sí	1	1	0		1	0	
	No	45	34	11		29	16	
Uso de drogas recreativas		45	34	11	No significativo	29	16	No significativo
	Sí	1	1	0		1	0	
	No	44	33	11		28	16	
Uso de preservativo			25	9	No significativo	23	11	No significativo
	Sí	33	24	9		22	11	
	No	1	1	0		1	0	
Infecciones de transmisión sexual		38	28	10	No significativo	25	13	No significativo
	Sí	7	4	3		3	4	
	No	31	24	7		22	9	
Cambio de tratamiento		41	32	9	No significativo	27	14	No significativo
	Sí	4	3	1		3	1	
	No	37	29	8		24	13	
Disminución del recuento de células CD4		43	33	10	No significativo	29	14	No significativo
	Sí	12	11	1		9	3	
	No	31	22	9		20	11	
Incremento de la carga viral		44	35	9	No significativo	29	15	No significativo
	Sí	14	11	3		7	7	
	No	30	24	6		22	8	

* Prueba exacta de Fisher para datos de recuento

## Discusión

La presente investigación describe los cambios genéticos y de resistencia molecular a la terapia antirretroviral experimentados por el HIV a los 12 y a los 18 meses de seguimiento en una cohorte de militares peruanos. Considerando el tiempo de análisis, presumimos que el virus podria haber experimentado cambios genéticos debido a factores intrinsecos (mutaciones generadas por errores de la transcriptasa inversa, sistema APOBC3 o «artefactos» del propio experimento) y a factores extrínsecos (factores socio-epidemiológicos, terapia antirretroviral) de la infección virus-huésped.

Si bien el estudio de los factores intrinsecos escapa de los objetivos de este estudio, no hemos detectado casos de hipermutación en las muestras analizadas. Además, hemos consignado el uso de la ADN polimerasa de alta fidelidad con el fin de evitar mutaciones derivadas de los procedimientos experimentales (artefactos).

Con relación a la determinación de si algún factor extrinseco podria estar relacionado con los cambios en la diversidad genética y en la resistencia, además de analizar las caracteristicas genéticas del HIV, hemos recabado información socio-epidemiológica sobre la conducta sexual de riesgo de la población militar seleccionada. De acuerdo con este análisis, se encontró evidencia de conducta bisexual, contacto con mujeres trabajadoras sexuales, uso inconsistente del preservativo y presencia de infecciones de transmisión sexual en más del 19 % de los participantes, lo que demuestra la existencia de conducta sexual de riesgo.

En concordancia con nuestros hallazgos, una reciente investigación reveló que en militares norteamericanos con HIV, el uso del preservativo también habia sido inconsistente (53 %) con una incidencia de infecciones de transmisión sexual similar a la de nuestro estudio (18,9 %) ^23^.

La incidencia de infecciones de transmisión sexual es consecuencia de una evidente conducta sexual de riesgo, la cual se ha demostrado que es más frecuente en la población militar que en la civil ^34^. Esto podría deberse a que en la población militar existen factores psicosociales como el abuso de alcohol, el trauma sexual militar, los trastornos de la salud mental ^23^^,^^24^, además de una fuerte presión colectiva por demostrar masculinidad, lo que, en su conjunto, conduce a un mayor número de parejas sexuales ocasionales e incremento de la frecuencia de infecciones de transmisión sexual ^25^.

La conducta sexual de riesgo también ocasiona la presencia de infecciones mixtas o superinfecciones con diferentes cepas de HIV ^35^, lo cual tiene un efecto adverso sobre el estado inmunológico del paciente ^36^^,^^37^^)^ y, posiblemente, sobre su respuesta a la terapia antirretroviral ^38^. Para evidenciar estos eventos, hemos comparado las caracteristicas genéticas de las cepas de HIV en dos intervalos de tiempo separados, al menos, por un año, con el fin de dilucidar si estos virus mantienen o no una relación filogenética a partir de un mismo paciente.

Nuestros hallazgos revelan que las cepas virales formaron grupos monofiléticos entre las variantes virales analizadas entre T_1_ y T_2_, lo cual sugiere ausencia de reinfección o superinfección mixta con nuevas variantes genéticas. Se observó un incremento del promedio de linfocitos CD4 desde T_1_ a T_2_, lo cual es consistente con la baja carga viral y la ausencia de casos de reinfección de cepas de HIV.

Si bien para determinar posibles superinfecciones de HIV se requieren mayores análisis moleculares como la realización de ensayos de movilidad de heterodúplex, clonación y secuenciación ^39^, o secuenciación de última generación ^40^, el análisis de secuencias mediante filogenia también puede ser de utilidad en la práctica de rutina para identificar conductas sexuales de riesgo ^41^, lo cual revela el potencial de la biología molecular para develar eventos de superinfecciones a pesar de no disponer de mayor información socio-epidemiológica.

Por otro lado, hemos demostrado que el subtipo B fue la variante predominante durante los dos tiempos de análisis. Esto es coherente con el hecho de que el subtipo B ha sido la principal variante que circula en el Perú desde hace más de 20 años ^9^^,^^42^. Sin embargo, también hemos detectado una nueva forma recombinante de tipo CRF02_AG (subtipo A para el gen *gag* y AG por el gen *pol),* la cual ha sido previamente reportada en otros países sudamericanos como Brasil, Ecuador, Venezuela y Argentina ^43^^-^^46^. Los análisis previos de esta variante revelaron una alta identidad con las cepas virales provenientes de la Guyana Francesa ^9^; sin embargo, se requiere continuar con la vigilancia de subtipos de HIV para determinar si el nexo epidemiológico de esta variante corresponde a uno o más paises de la región, reforzando que el ingreso de estas variantes está vinculado a la movilidad internacional o a contactos con redes sexuales amplias, reiterando la necesidad de fortalecer la vigilancia molecular en la población militar, dado su frecuente desplazamiento geográfico.

Asimismo, hemos llevado a cabo el análisis de la selección evolutiva de las muestras estudiadas mediante la determinación de los cambios a nivel de polimorfismo genético. Para ello, hemos utilizado el test de Tajima con el fin de calcular D, cuyos valores negativos sugieren la presencia de eventos de cuello de botella ^33^, mientras que la presencia de valores dN/ dS menores de 1 detectada en la población (cuadro 2) sugiere un proceso de presión de selección purificadora ^47^. Si bien D mostró valores negativos, lo que sugiere un aparente cuello de botella, hemos recurrido al análisis de los datos socio-epidemiológicos y clinicos de los participantes para obtener información relevante al respecto.

En primera instancia, se sabe que el 100 % de los participantes llevan recibiendo terapia antirretroviral por largos periodos (entre 10 y 20 años, no se presentan los datos) con favorable supresión de la carga viral. Esto sugiere que los periodos prolongados de exposición a la terapia antirretroviral podrian estar favoreciendo la reducción de la carga viral y, concomitantemente, la presencia de eventos de cuello de botella en HIV. De hecho, se ha demostrado que, mediante el uso del raltegravir en ensayos *in vitro,* se promovió la diversidad genética de cepas de HIV, con subsecuente reducción del tamaño efectivo de la población viral y cuello de botella ^48^.

Por otro lado, el análisis de un grupo de pacientes con menos de 1 año de terapia antirretroviral reveló la ausencia de eventos de cuellos de botella en HIV-1 y que la diversidad genética habia sido nula o minima en las regiones *gag-pro-pol*^49^. Estos datos en su conjunto revelan que, tanto la acción del medicamento antiviral, como el periodo o intervalo de tiempo que toma el virus para evolucionar, podrian ejercer conjuntamente una presión de selección sobre las variantes de HIV analizadas en este estudio.

Con relación a esto último, hemos demostrado diferencias significativas (p < 0,05) en la diversidad genética del HIV analizado en T_1_ frente a T_2_ en ocho muestras a los 12 y a los 18 meses de seguimiento. Nuestros hallazgos contrastan con otro estudio que reveló que, a partir de un grupo de 20 sujetos con terapia antirretroviral continuo, el HIV no habia experimentado ningún cambio significativo en su diversidad genética a lo largo de cinco años de seguimiento ^50^.

Si bien algunas consideraciones de diseño metodológico y experimental podrian influir en los resultados obtenidos, también es importante considerar la presencia de uno o más factores externos que podrian haber ejercido una presión de selección del HIV, incrementando la variabilidad genética de los genotipos virales analizados en el presente estudio.

En nuestro análisis de prevalencia de mutaciones de resistencia, en función de los casos de HIV que presentaron cambios significativos de nucleótidos (cuadro 4), se logró identificar que en estas cepas virales se presentó una mayor frecuencia de mutaciones de resistencia y cambios en el perfil de resistencia en comparación con aquellas variantes que no presentaron cambios significativos. Estos hallazgos sugieren que la terapia antirretroviral habria generado cambios en el perfil de resistencia de los participantes mediante la selección de mutaciones puntuales, afectando la variabilidad genética del HIV, hecho que habria ocurrido inmediatamente después de T_2_. Es importante señalar que, con relación a este último punto, solo el 25 % de los sujetos cambiaron de terapia antirretroviral (cuadro 1), lo que sugiere que otro factor externo adicional habria influido sobre la presencia de estos cambios.

Al respecto, nuestro análisis de variables socio-epidemiológicas reveló que la variable asociada con el cambio de perfil de resistencia del HIV fue la edad (principalmente sujetos mayores de 39 años). Resulta interesante que los ocho casos asociados con un cambio significativo de la variabilidad genética del HIV entre T_1_ y T_2_ se presentaron en pacientes con edades superiores a los 39 años (no se presentan los datos). Estos hallazgos sugieren que el cambio de perfil de resistencia del HIV en la población estudiada no se ajustó necesariamente con el cambio de esquema terapéutico, sino que podria asociarse con factores subyacentes circunscritos con la edad del sujeto.

Asi pues, es posible que la farmacocinética de algunos antirretrovirales haya estado parcialmente alterada por la edad, tal como se observó con el tenofovir, cuya vida media en el ambiente intracelular fue menor en los pacientes pediátricos y en los jóvenes que en la población de adultos, lo que revela una mayor concentración del fármaco a nivel plasmático y una menor tolerabilidad renal en adultos ^51^. Esto también podria explicar la baja adhesión y concomitantemente el desarrollo de la resistencia a los antirretrovirales.

Por tanto, la efectividad del tenofovir en esta población de adultos militares podria haberse visto alterada por una baja adhesión debido a alta exposición, toxicidad renal y presentación de sintomas, razón por la cual, algunos autores recomiendan que la administración de este fármaco se dé bajo vigilancia renal en mayores de 65 años ^52^^-^^55^.

Además, casi la mitad de la población de militares se caracterizó por tener entre 11 y 20 años de haber sido diagnosticados con infección por HIV (cuadro 1), con una media de 12 años de diagnóstico para los sujetos mayores de 39 años (no se presentan los datos). Este tiempo de infección, sumado al tiempo de tratamiento, sugiere que el ADN proviral de los sujetos analizados podria haber sufrido cambios relacionados con la diversidad genética y la resistencia a la terapia antirretroviral. Al respecto, se ha demostrado que, durante el transcurso del tiempo, el ADN proviral en los sujetos con terapia antirretroviral se mantiene más estable y persiste en el ADN del huésped, en comparación con aquellos sin terapia antirretroviral, en quienes la replicación activa del virus genera un ambiente menos favorable para la estabilización del provirus y lleva a su degradación ^56^.

Asimismo, un estudio demostró que, a pesar de que la carga viral se logra mantener a bajos niveles, el virus continúa evolucionando, afectando de manera importante su variabilidad genética a nivel de cuasiespecies ^57^. Estos datos en conjunto podrian explicar por qué el ADN proviral de estos individuos con mayor tiempo de diagnóstico y tratamiento es más variable, lo cual se relaciona también con los cambios en el perfil de resistencia según el tiempo.

Con relación a la población civil peruana con HIV, se ha documentado que la diversidad genética del virus y la aparición de resistencia están más estrechamente relacionadas con las interrupciones de la terapia antirretroviral, la falta de adhesión y el uso previo de esquemas subóptimos, revelando frecuencias relevantes de resistencia transmitida y adquirida que se asocian con la historia de exposición a antirretrovirales, con brechas de adhesión y manejo clinico ^7^^,^^58^. Asimismo, los estudios clinicos de pacientes con falla virológica en el Perú han demostrado que la resistencia frecuentemente se asocia con los años de tratamiento, los cambios previos de esquema y las demoras en el cambio de terapia tras la falla virológica ^58^^,^^59^.

Sin embargo, en nuestro estudio, en el que la población militar presentó evidente supresión virológica, el HIV también presentó cambios genéticos significativos asociados con la edad. Esta diferencia sugiere que el HIV que afecta este tipo de población puede presentar patrones evolutivos particulares debido a la historia de terapia antirretroviral más prolongada, mayor estabilidad en los esquemas terapéuticos, menor interrupción del tratamiento, pero mayor prevalencia de factores de riesgo conductuales y psicosociales que afectan la adhesión y la exposición al HIV ^59^^,^^60^.

Además, los hallazgos de este estudio muestran evidencias virológicas a nivel molecular que podrian ser relevantes para proponer un mejor manejo clinico de la infección por HIV tales como: 1) vigilancia más estrecha en los mayores de 39 años, considerando a la edad como un factor predictor temprano de los cambios en la resistencia; 2) monitoreo renal rutinario, principalmente porque los antirretrovirales (como el caso de tenofovir) muestran aumentos de la exposición cuando disminuye la función renal; 3) evaluación personalizada de adhesión a la terapia antirretroviral, principalmente en los sujetos mayores que 39 años; y 4) incorporación de la vigilancia molecular longitudinal mediante la genotipificación periódica, contribuyendo a la anticipación de fallas terapéuticas y a diseñar cambios de esquema basados en evidencia genotípica y clínica ^54^^,^^55^^,^^61^^-^^63^.

En conclusión, hemos demostrado que el HIV que infecta militares que han recibido por largo tiempo terapia antirretroviral y con carga viral suprimida, presenta eventos de cuello de botella y diversidad genética, cuya diferencia de nucleótidos entre un tiempo y el otro llega a ser significativa, principalmente en aquellos casos en los que se observaron cambios del perfil de resistencia a los antirretrovirales, la cual se asocia con un incremento en la edad del sujeto.

En esa misma linea, la evidencia disponible apoya que la edad (mediante la disminución de la función renal y los cambios farmacocinéticos de los antirretrovirales) contribuye a modular la exposición a antirretrovirales y, por ende, a la selección de mutaciones de resistencia.

Cabe resaltar que la mayoria de los estudios revisados documentan efectos de farmacocinética clinicamente relevantes en edades más avanzadas que las analizadas en nuestra cohorte; sin embargo, nuestros hallazgos también sugieren que existe un papel combinado de edad, tiempo acumulado de terapia antirretroviral y factores clinicos y conductuales, que podrian alterar estos efectos, y que, en consecuencia, deberian ser considerados de aqui en adelante, para un mejor manejo clinico del paciente.

Por otro lado, es importante señalar que una de las principales limitaciones de este estudio fue el reducido tamaño muestral, por lo tanto, los resultados no pueden ser considerados representativos para la población militar peruana con HIV. Sin embargo, el diseño longitudinal sobre las caracteristicas genéticas del HIV ha permitido hallar evidencias socio-epidemiológicas, clinicas y moleculares que podrian ser útiles para mejorar las estrategias de la terapia antirretroviral en función del tiempo, principalmente para el monitoreo de la resistencia por medio del perfil genotipico del virus.

Asimismo, se ha logrado caracterizar la evolución del HIV mediante la identificación de los subtipos y las formas recombinantes emergentes. Además, se consiguió hacer el seguimiento de la resistencia a los antirretrovirales a lo largo del tiempo, lo que podria servir como antecedente para futuros estudios enfocados en otros grupos con diferente comportamiento sexual de riesgo.

Dada la escasez de estudios previos en población castrense con HIV, los hallazgos constituyen una contribución significativa para determinar los factores que promueven la evolución molecular del HIV, y su impacto sobre la salud de estos pacientes.
